# Experimental study of true triaxial high pressure subcritical water impact fracturing

**DOI:** 10.1038/s41598-024-51189-2

**Published:** 2024-01-11

**Authors:** Shaobin Hu, Xiaofei Wang, Enyuan Wang

**Affiliations:** 1 Tunnel and Underground Engineering Institute, College of Civil and Transportation Engineering, HoHai University, Nanjing, 210098 Jiangsu China; 2 School of Safety Engineering, China University of Mining and Technology, Xuzhou, 221116 Jiangsu China

**Keywords:** Hydrology, Solid Earth sciences, Engineering

## Abstract

A new fluid alternative to slick water for fracturing shale gas can reduce the waste of water resources and improve the extraction efficiency, enabling volumetric fracturing. For the new fracturing technique, the experiments of different release pressures under pre-injection and for pre-injection were conducted using a self-designed true triaxial experimental system, and the pressure pulse curves were plotted to analyze the fracturing principle. The experimental results showed that: (1) the pressure rise curve in the reactor can be divided into five stages: initial reaction, linear pressure rise, rate slowdown, instantaneous pressure release, and residual pressure stages; (2) Pre-filling fracturing requires a smaller expansion ratio, weaker pressure degradation, resulting in better fracturing effect; (3) The increase in the initial fracture length leads to an increase in the pressure required to extend the fracture, and high-pressure subcritical water impact fracturing achieved fracture extension at a lower fluid pressure; (4) The fractal dimension has a strong linear relationship with fracture complexity, which is a new option when evaluating the fracturing effect. Volumetric fracturing allows for the creation of more tiny trenches that increase reservoir permeability, leading to better recovery of the reservoir’s energy resources.

## Introduction

Shale gas is widely distributed worldwide and has great potential for exploitation^1,2^. Shale gas accumulations have low porosity and extremely low matrix permeability^3,4^. Hydraulic fracturing is the main means of shale gas extraction. Currently, engineers in North America have developed an extraction technique based on horizontal well casing termination, cluster injection, and large-scale slip fracturing and simultaneous fracturing of water sections. This novel fracturing technique (volumetric fracturing) has greatly improved the recovery rate and production of shale gas^5,6^. Many scholars conducted experimental studies and field application tests on hydraulic fracturing of shale gas, and the research results are of significance for the improvement of the technique^7–10^.

To improve fracturing efficiency and form a novel fracturing technique system, many scholars have proposed new alternative fluids to fracturing fluids, including CO_2_^11,12^, liquid nitrogen^13^, and high-energy gas^14^. The reduction of water use can solve the challenges of shale gas extraction in arid and water-scarce regions, while preserving precious water resources^15,16^. Advantages of supercritical CO_2_ fracturing are: a low fracture initiation pressure, complex fracture-causing fractures, and easier communication of microfractures^17,18^; three aspects of fracture initiation pressure, fracture distribution, and microfracture extension are currently studied experimentally^19–21^; fracture extension was observed by CT, scanning electron microscopy, and acoustic loss measurement instruments^22,23^. The volume expansion and freezing of liquid nitrogen as it changes from the liquid phase to the gaseous state produces multiple microfractures, giving it an advantage when used during fracturing^24^. Nitrogen is widely available and has the potential to replace water as a fracturing fluid^25,26^. Numerous scholars have studied its fracturing characteristics and found that: phase change stress and temperature stress act together in fracture extension^27^. Zhai et al. established a model for liquid nitrogen fracturing and applied it to the extraction of coalbed methane to obtain efficient fracturing effect^13^. The supercritical CO_2_ and liquid nitrogen fracturing techniques are unable to produce pulse impacts during the fracturing process. The high-energy gas fracturing technique generates shock waves to fracture the rock after instantaneous release^28,29^. Zhu et al. explored the application of high-energy gas fracturing in wellbore drilling, and its fracture characteristics were different from conventional hydraulic fracturing, supercritical CO_2_ fracturing, and liquid nitrogen fracturing. This is because it produced circumferential fractures, which made fracturing more effective^30,31^. More fracture channels were more favorable for the resolution and extraction of shale gas^32,33^. However, the shock waves generated by high-pressure gas can cause damage to the original structure of the wellbore and create engineering difficulties^34,35^.

Conventional hydraulic fracturing was improved while simultaneously increasing the water temperature and pressure to reach a subcritical state^36,37^, and the instantaneous impact can achieve the effect of volumetric fracturing. We proposed a novel volumetric fracturing technique: high-pressure subcritical water fracturing. It works by instantaneously releasing a large amount of heat from a hydrothermal agent under detonator ignition to heat the water inside the vessel to make its pressure and temperature rise rapidly until it reaches the release pressure (more than 30 MPa) and then instantly releases and fractures the rock body. This research undertook exploratory experiments on this technique, including: (1) establishing a true triaxial high-pressure hydrothermal fracturing experimental system; (2) analyzing the test pressure curves at different release pressures; (3) studying the effect of whether or not water is pre-filled as evinced by local pressure curves.

## Materials and methods

### Preparation of specimens

For this experiment, C100 special high strength cement with a water to ash ratio of 0.115 was used, mixed and poured into a mold. The molds filled with cement were placed on a vibrating table and vibrated to reduce the porosity and ensure the uniformity of the specimens. The use of cement as a substitute material eliminates the errors in experimental results caused by anisotropy arising from the naturally occurring primary fractures and uneven distribution of constituent minerals in the rock. The use of rock specimens in the study of the effect of rock anisotropy on fracture by some scholars can be of interest for future work.

**Figure 1:**
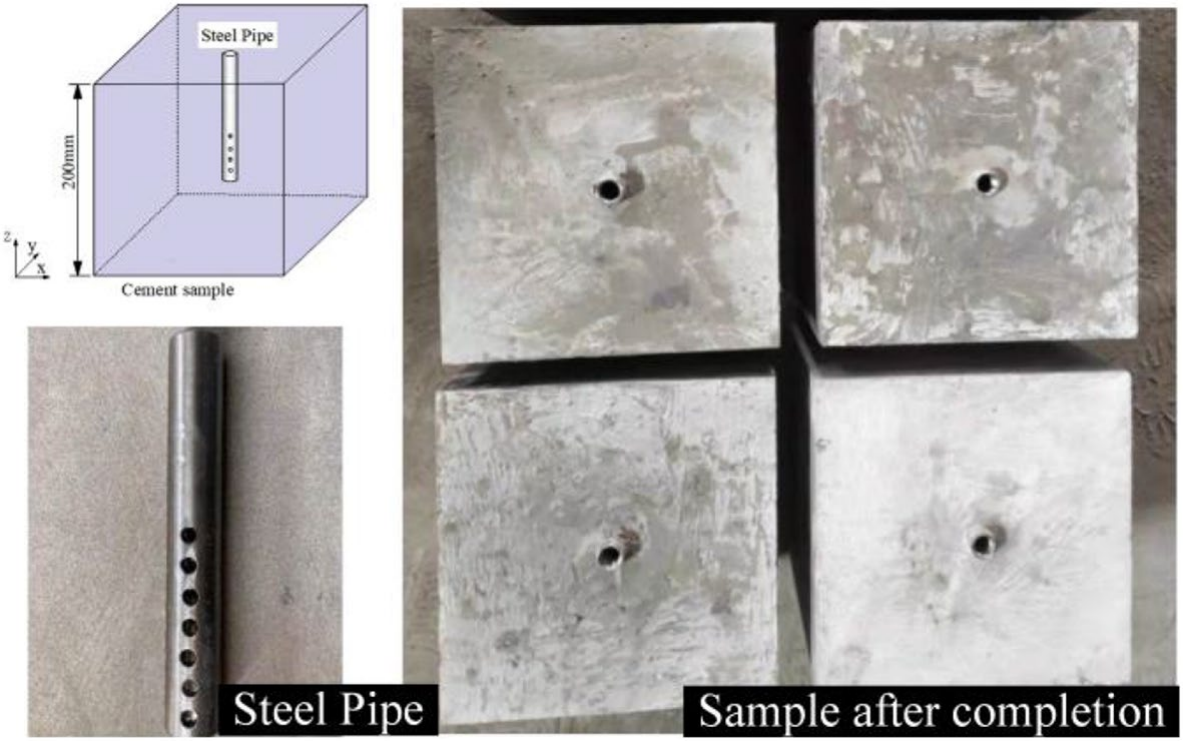
Schematic diagram of specimen fabrication.

The fracturing tube is a custom-made stainless-steel tube with the following parameters: length 110 mm, outer diameter 15 mm, inner diameter 11 mm, perforated section length 50 mm, four rows of holes, seven perforations per row (hole diameter 5 mm) and ¼-in. (6.25 mm) internal threads at the end, as shown in Fig. 1. The fracture tube was inserted into the shaken cement slurry and left to stand for 24 h before being demolded (forming the borehole model). After demolding, the specimens were conditioned for 14 d to ensure that they reached their calibrated strength. The prepared specimens were subjected to tests to determine their mechanical parameters (Table 1).

**Table 1:** Physical parameters of the experimental specimens.

Density /(g/cm^3^)	Compressive strength/MPa	Tensile strength/MPa	Permeability/md	Modulus of elasticity/GPa	Poisson's ratio	Fracture toughness/ (MPa*m^1/2^)
2.35	105	9.6	46.4	22.6	0.14	5.0

### Experimental system

**Figure 2:**
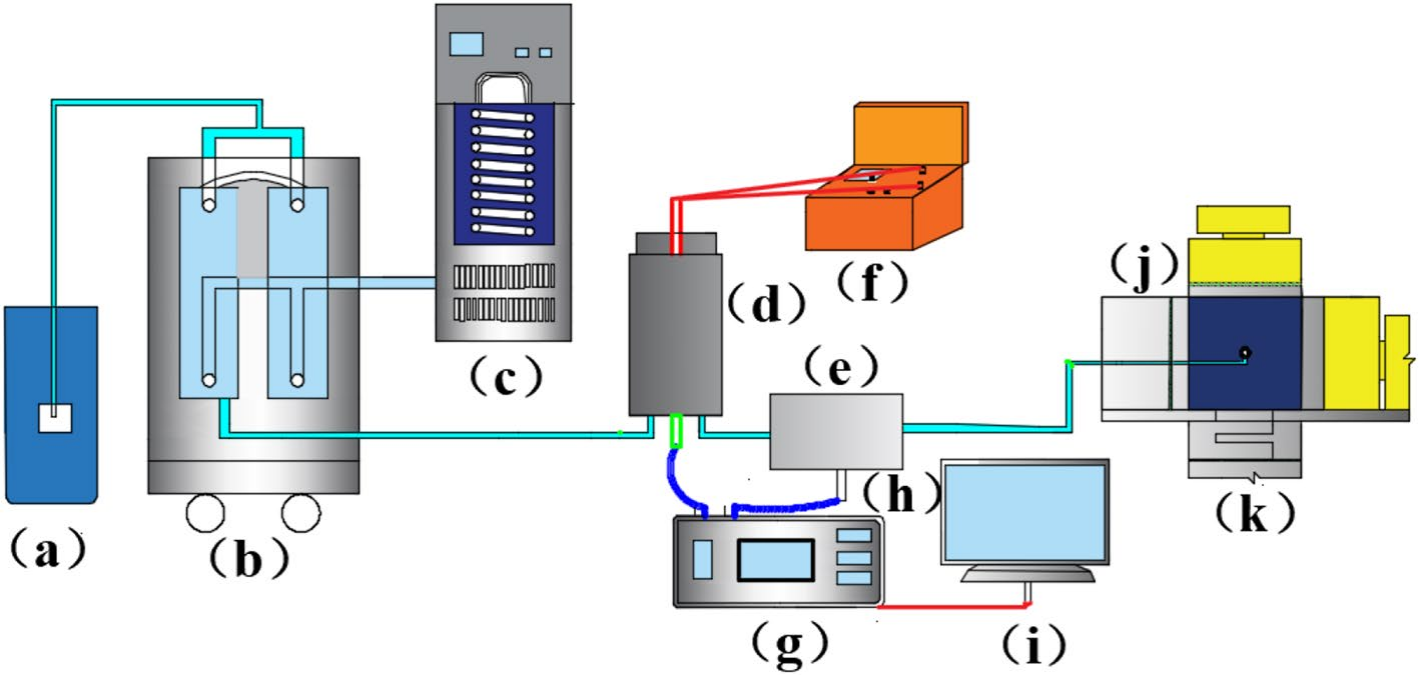
Schematic diagram of the test system (a, water tank; b, double-cylinder pump; c, water bath system; d, hydrothermal reactor; e, oil pressure valve; f, detonator; g, data acquisition instrument; h, pressure sensor; i, computer; j, oil pressure pump; k, true triaxial loading system).

**Figure 3:**
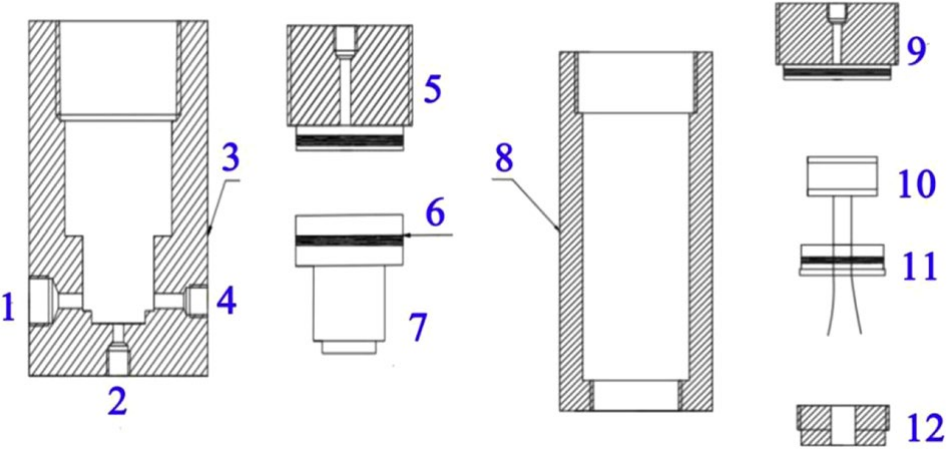
Structure of the hydrothermal reactor and oil pressure valve (1.Pressure sensor interface 2. Water inlet 3. Oil pressure valve body 4. Water outlet 5. Oil inlet end 6. Sealing O-ring 7. Moving copper block 8. Reactor body 9. Reactor outlet 10. Polyenergy agent 11. Sealing cover 12. Plugging block).

**Figure 4:**
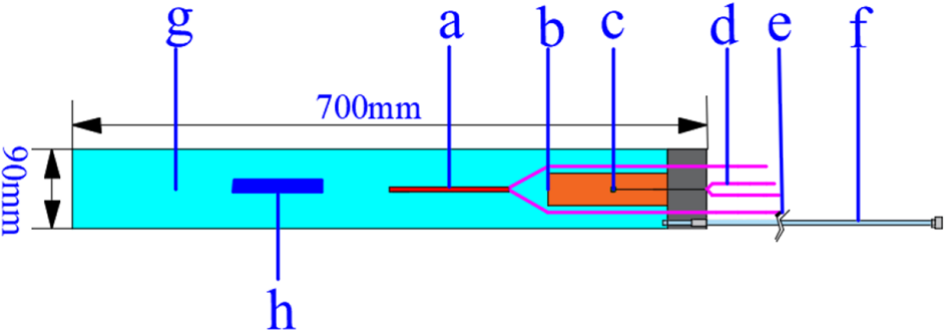
Schematic diagram of the hydrothermal fracturing cylinder structure (a, heating rod; b, hydrothermal fusion agent; c, ignition charge; d, excitation wire; e, heating wire; f, safety valve; h, weak bar).

A true triaxial high-pressure hydrothermal fracturing experimental system was designed, which consists of three main components: a high-pressure hydrothermal reactor system, a true triaxial loading system, and a pressure acquisition system. The high-pressure hydrothermal reactor system consists of a high-pressure hydrothermal reactor and a pressure relief switch (Fig. 2). The high-pressure hydrothermal reactor system was made, which is composed of a high-pressure hydrothermal reactor and a pressure relief switch (Fig. 3). The pressure relief switch uses an oil pressure pump to provide oil pressure to close the fluid outlet, when the switch needs to be opened simply open the oil pressure valve to remove the oil pressure. The true triaxial loading system uses an oil pressure pump and stainless-steel gaskets to achieve true triaxial perimeter pressure loading. The pressure acquisition system consists of a pressure transducer, a data acquisition instrument and a computer. The pressure transducers are cyg140f (0–60) and cyg140g (0–100) types with ranges of 0–60 MPa and 0–100 MPa respectively and a response frequency of 20 kHz–2.0 MHz. The 0.25-MHz mode was selected for the experiment. A new type of hydrothermal cracking cylinder was developed (Fig. 4), which can generate a high-temperature and high-pressure hydrothermal shock wave after excitation to crack the rock.

### Experimental procedure

A total of six sets of experiments (S1–S6) were conducted, and different release pressures were controlled through the pressure switch to observe the effect of release pressure on the fracturing effect. To emulate the geopathic stress in the mine, the confining pressures were controlled at 7 MPa, 5 MPa, and 4 MPa in different experimental groups of release pressures. In groups under different confining pressures, due to the delayed opening and closing of the pressure switch and the extremely fast hydrothermal reaction speed, it was difficult to control the release pressure, and after testing we can only guarantee a general range of release pressures. Although the details are different in all experiments, the experimental procedure was as follows:

1. System and specimen connection: the test system was connected, the pressure transducer was connected to the pipeline through a specially machined fitting, and the prefabricated stainless-steel tube in the specimens was joined to the pipeline through a threaded connection;
2. Three-axis circumferential pressure loading: after the connection of the experimental system was completed, the specimens were placed on the three-axis loading table, the position of the stainless-steel spacer was adjusted, and the pressure loading was achieved through a manual hydraulic pump, and the three axes needed to be loaded simultaneously in the test;
3. Pre-injection: a double-cylinder pump was used to inject water into the pipe, the flow rate was set to 40 ml/min, then we waited until there was pressure indication to turn off the double-cylinder pump to achieve pre-injection;
4. Closed pressure switch: the oil pressure was loaded to 60 MPa by a manual oil pressure pump to achieve closure of the oil pressure switch;
5. Excitation: The ignition head in the polyenergy agent was excited by the detonator to ignite the polyenergy agent and the water pressure increased rapidly;
6. Pressure release: observed through the sensor pressure in the hydrothermal reactor, after reaching the required pressure, the hydraulic pump oil pressure was removed, the pressure switch opened, high-pressure water instantly released fracturing specimens;
7. Removing the specimens and spraying paint: while unloading the true triaxial pressure at the same time, the specimens were removed, the better to observe the fracture morphology, then white paint was sprayed evenly on the surface of the specimens before photographing it.

## Results and discussion

### Analysis of the experimental pressure curve

**Figure 5:**
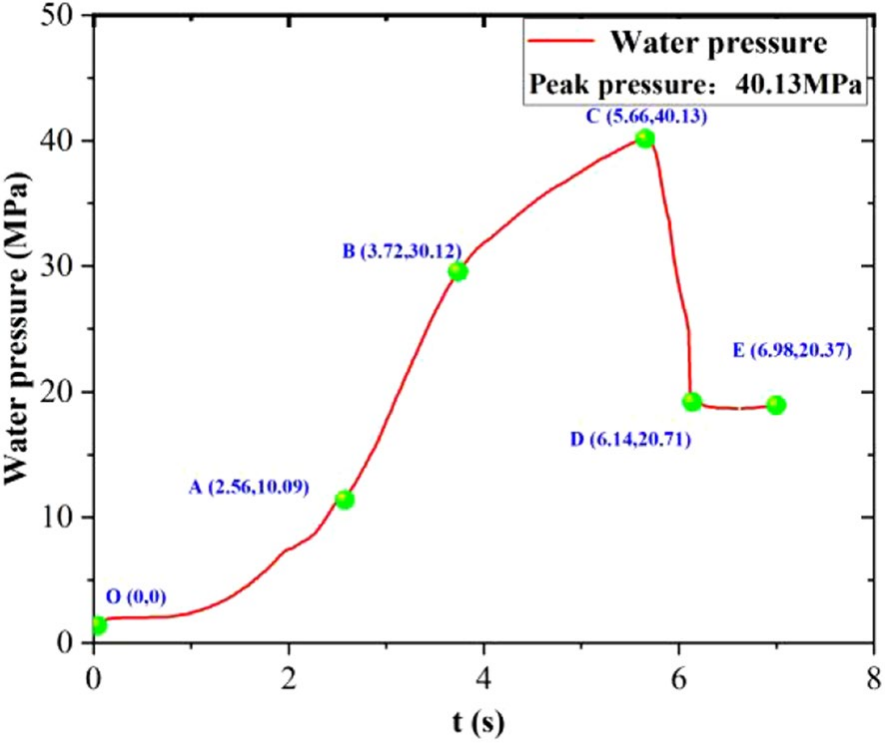
Pressure curve in the reactor without pre-injection of water 40.13 MPa.

The curve in Fig. 5 shows that the pressure evolution can be divided into five stages, which are: initial reaction stage, linear pressure increase stage, rate slowdown stage, instantaneous pressure release stage, and residual pressure stage.

OA section: from excitation to 2.56 s; the pressure was increased from atmospheric pressure (0.1 MPa) to 10.09 MPa. The gradual increase in the rate of pressure increase in this phase was attributed to the increase in the amount of drug involved in the reaction after excitation, the reaction rate of the agent increased, and the rate of heat generation increased; the heat was transferred to the water, which made the water expand and the increased the pressure faster.

AB section: from 2.56 to 3.72 s; the pressure increased from 10.09 to 30.12 MPa; this stage is the fastest phase of fluid pressurization, the pressure increased linearly, the heating agent reaction rate reached its peak at this stage, and the peak remained at 0.71 s.

BC section: in this stage the pressurization was maintained, but the pressurization rate gradually decreased to about 0 MPa/s. The heating agent continued to supply heat at this stage, but as the reaction proceeds the agent was consumed, the reaction rate gradually decreased, corresponding rate of heat generation also gradually decreasing, and the fluid pressure increasing more slowly. From 3.72 to 5.66 s, the pressure increased from 30.12 to 40.54 MPa.

CD section: the pressure decreased at the moment of release. The pressure dropped from a peak of 40.13 MPa to 20.71 MPa; after release of the pressure, the fluid was ejected from the outlet of the oil-pressure valve, and the heating could be considered to have stopped because the fluid flowed out of the heating agent and was separated therefrom; the fluid pressure dropped rapidly after communicating with the pressure in the gas circuit (saturation temperature drop). As the temperature of the fluid at this point was far higher than the saturation temperature at the corresponding pressure, the fluid underwent violent boiling and rapid vaporization.

DE section: the pressure in this stage was maintained at about 20 MPa. This stage can reflect the crack extension, the residual pressure was greater than the static crack-initiation pressure, then the crack extension could be achieved, and vice versa, the crack stopped. The pressure could be maintained at a stable level due to the specimens being subject to a high circumferential pressure and the small amount of water seeping out of the fracture.

**Figure 6:**
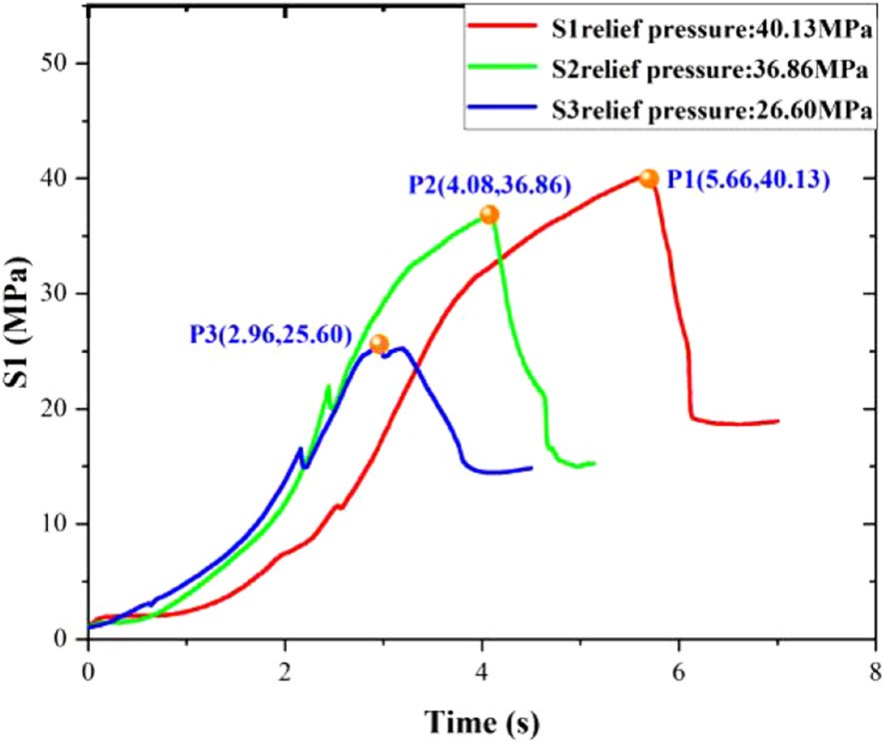
Pressure curve of three groups of experimental reactors without pre-injection of water.

Figure 6 shows pressure curves in three groups of experimental reactors without pre-injection of water; the peak pressures were 40.13 MPa, 36.86 MPa, and 25.60 MPa, respectively. All the curves showed similar trends, and the peak pressures were changed by controlling the pressure-relief time. The residual pressure was between 15 and 20 MPa, and the residual pressure increased with increasing peak pressures.

**Figure 7:**
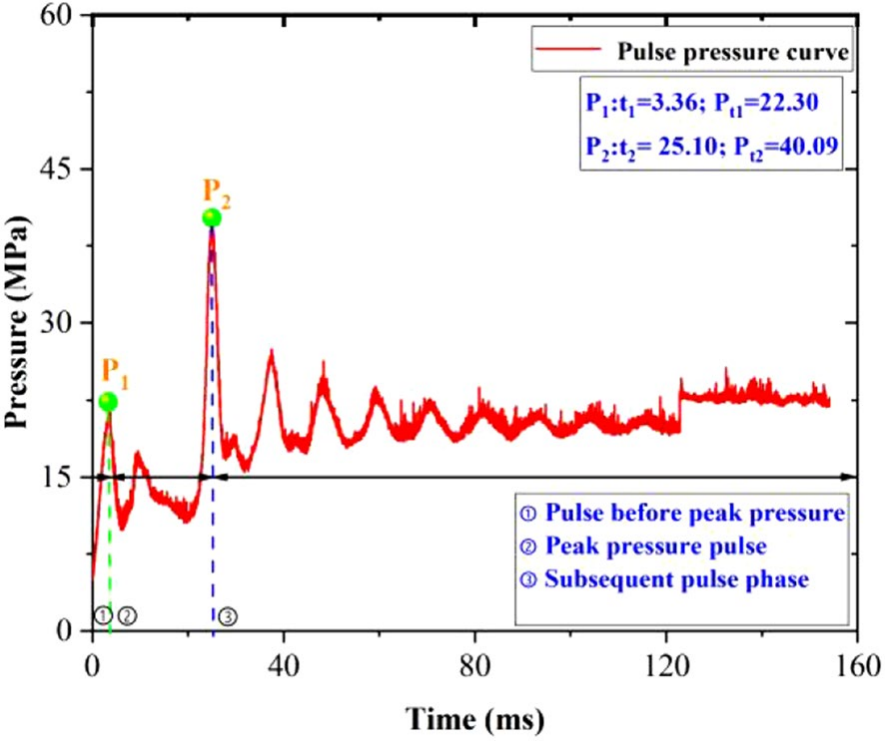
40.13 MPa relief oil pressure valve outlet pressure curve.

At 40.13 MPa the pressure curve at the outlet of the released oil pressure valve is as shown in Fig. 7. The pressure curve changed over three phases.

The first pulse phase, the pulse before the peak pressure. The reason for the pressure wave before the peak pressure was due to the mechanical piston type structure (with seal) inside the hydraulic valve, the actual opening process piston to move over one period of travel, the process takes a certain amount of time. The moment the piston moved (oil-pressure valve partially open), part of the high-pressure water vapor driven by the pressure difference into the oil-pressure valve gas chamber, the pressure at the outlet increases, the first wave peak; but as the piston continues to move (oil-pressure valve continues to open), due to the volume increasing, the fluid expansion gives rise to a phase change (liquefaction), the pressure rapidly decreases, and a wave trough appears.

The second pulse phase, the peak pressure pulse. The reason for the pulse fluctuation at this stage was that as the high-temperature, high-pressure water continued to flow into the oil-pressure valve gas compartment, filling the oil-pressure valve gas compartment chamber, the impact pressure acting pipeline liquid column, the pressure rose to reach the peak pressure (slightly less than the high-pressure hydrothermal generator release pressure). Due to the incompressibility of liquid water, the force transfer effect was excellent, and the impact pressure was transferred through the liquid column to act on the specimen hole wall, and the specimens were cracked, resulting in cracks. Crack expansion and volumetric expansion lead to pressure decay.

When the pressure dropped below the generator pressure, the high-temperature and high-pressure water continued to replenish the fracture holes and cracks in the specimens under the effect of pressure difference, and the pressure increased. As the process continues, the pressure stabilizes around the residual pressure (the pressure fluctuates slowly in this stage). In addition, because the fracture did not communicate with the external atmospheric pressure over a large area, there remained high-pressure water sequestered in the specimens under high perimeter pressure. If the record continues, the pressure drops after the water temperature drops, and when it is lower than the pressure required for crack extension, the crack no longer expands.

**Figure 8:**
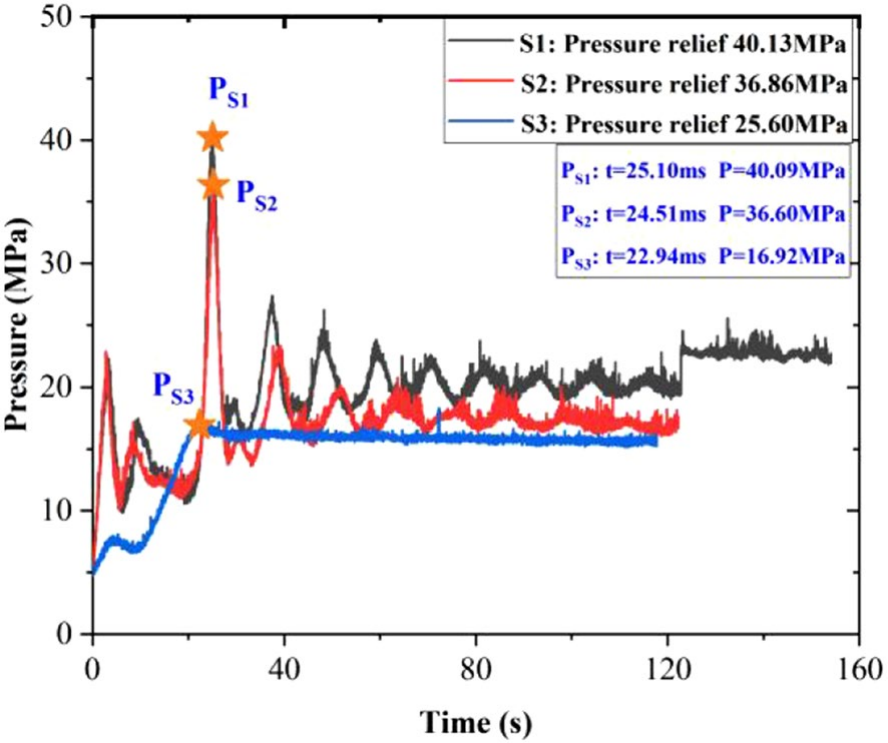
Without pre-injection of water three groups of experimental oil pressure valve port pressure curve.

Figure 8 demonstrates the pressure curves of the hydraulic valve port with different release pressures. The pressure curves of the hydraulic valve port were similar for the three experiments, and the corresponding peaks were 40.14 MPa, 36.55 MPa, and 25.48 MPa, respectively; comparing the data with the pressure curves in the reactor, the peaks of the curves of the hydraulic valve port were found to be consistent, and the peaks of the pressure curves of the hydraulic valve port decreased with the decrease of the release pressure. As the pressure relief pressure decreased, the time of pressure peak was extended from 11.56 to 16.02 ms. Comparing the residual pressure, it was found that the residual pressure of S3 was smaller than that of S1 and S2, indicating that the residual pressure in the specimen fracture was smaller.

**Figure 9:**
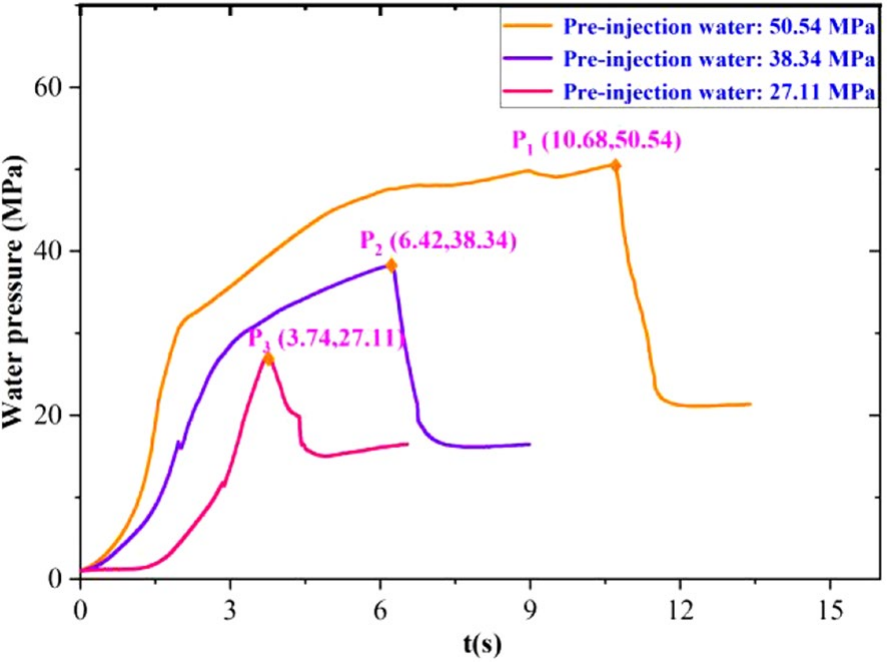
Pre-injection of water three groups of experimental reactor pressure curve.

Figure 9 shows the pressure curves of the experimental reactor for the three groups of pre-injected water, and the experimental pressure peaks were: 50.54 MPa, 38.34 MPa, and 27.11 MPa. All pressure curves were divided into the same five stages as those of the experimental group without pre-injected water. The experimental participation pressure was around 20 MPa, and the residual pressure tended to decrease slowly due to the triaxial confining pressure.

**Figure 10:**
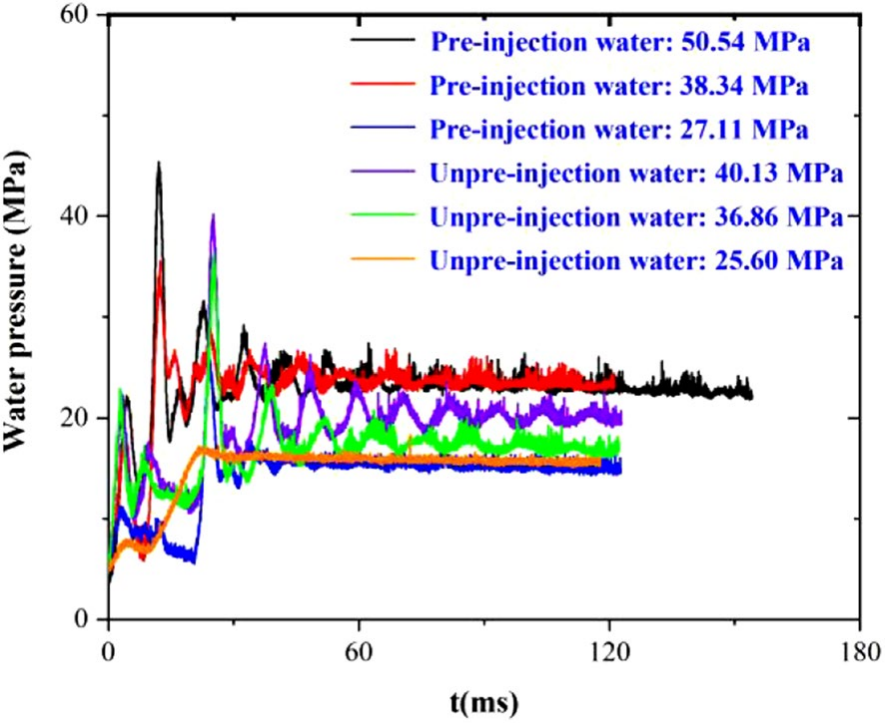
6 groups of experimental oil pressure valve port pressure curve.

Figure 10 demonstrates six groups of experimental oil pressure valve port pressure curve. All pulse curves can be roughly divided into three stages and the second peak after 25.60-MPa pressure relief without water injection did not appear significantly lower pressure. This is because the lack of pre-injection of water led to insufficient expansion of the second peak, which is also the common point of the S4–S6 pressure curve.

### Fractal morphology and calculation

**Figure 11:**
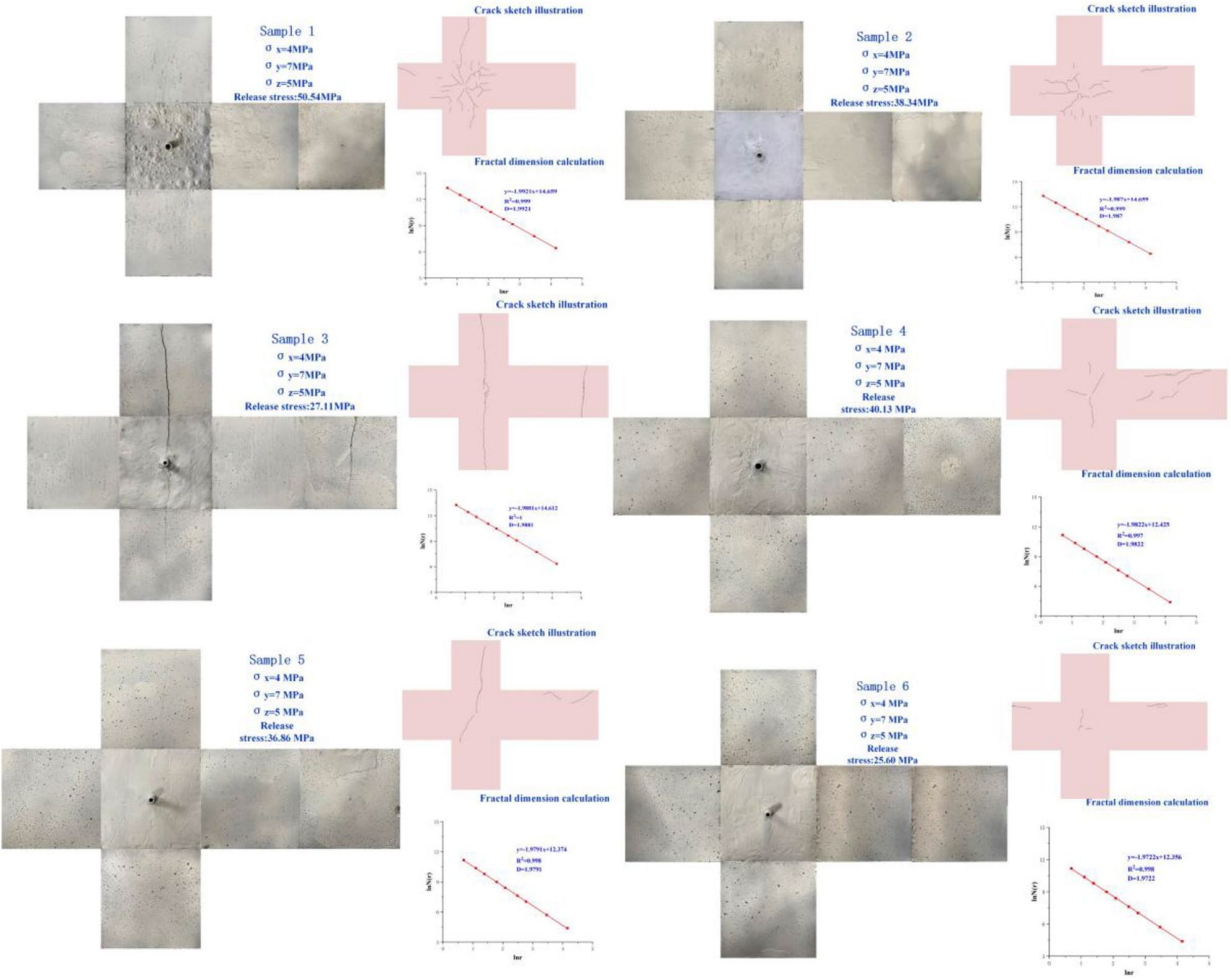
Six groups of experimental specimens fracture distribution extracted from fractal calculation.

After taking photographs of the specimens, the images were stitched together and due to the small size of the fractures relative to the specimens, the fractures were traced and drawn using Photoshop image-processing software to facilitate subsequent observations and analysis of the distribution pattern of the fracture morphology. The fracture distribution maps and fractal dimension calculations for Specimens S1-S6 are shown in Fig. 11.

Fracture complexity is important in evaluating the effect of fracturing as it provides a quantitative description of fracturing. Chen et al. defined a dimensionless quantity fc that overcomes the effects of different specimen sizes^23^. It is defined as:

1 $$f_{c} = \frac{{l_{total} }}{4d}$$

where: *l*_total_ is the total length of the induced fracture trace on the specimen surface, mm; *d* denotes the specimen edge length. By numerical analysis of the sample images, *l*_total_ can be obtained. in this study, it is the total length of fracture on the specimen surface.

Structural cracks are often analyzed fractally using the box-counting method^38,39^, which is implemented as follows: the entire crack distribution area is covered with a square grid (whose side length is *r*), the number of all grids containing cracks *N*(*r*) is counted, and the above calculation is repeated after changing the grid size *r* to obtain multiple sets of *r versus N*(*r*) data, and, if the ln(1/*r*) to ln*N*(*r*) relationship is linear relationship, then the cracks conform to the fractal characteristics. The box-counting method is generally governed by the following equation;

2 $$\ln \;N\left( r \right) = D\ln \left( \frac{1}{r} \right) + K$$

where: *K* represents a constant and *D* is the fractal dimension.

**Figure 12:**
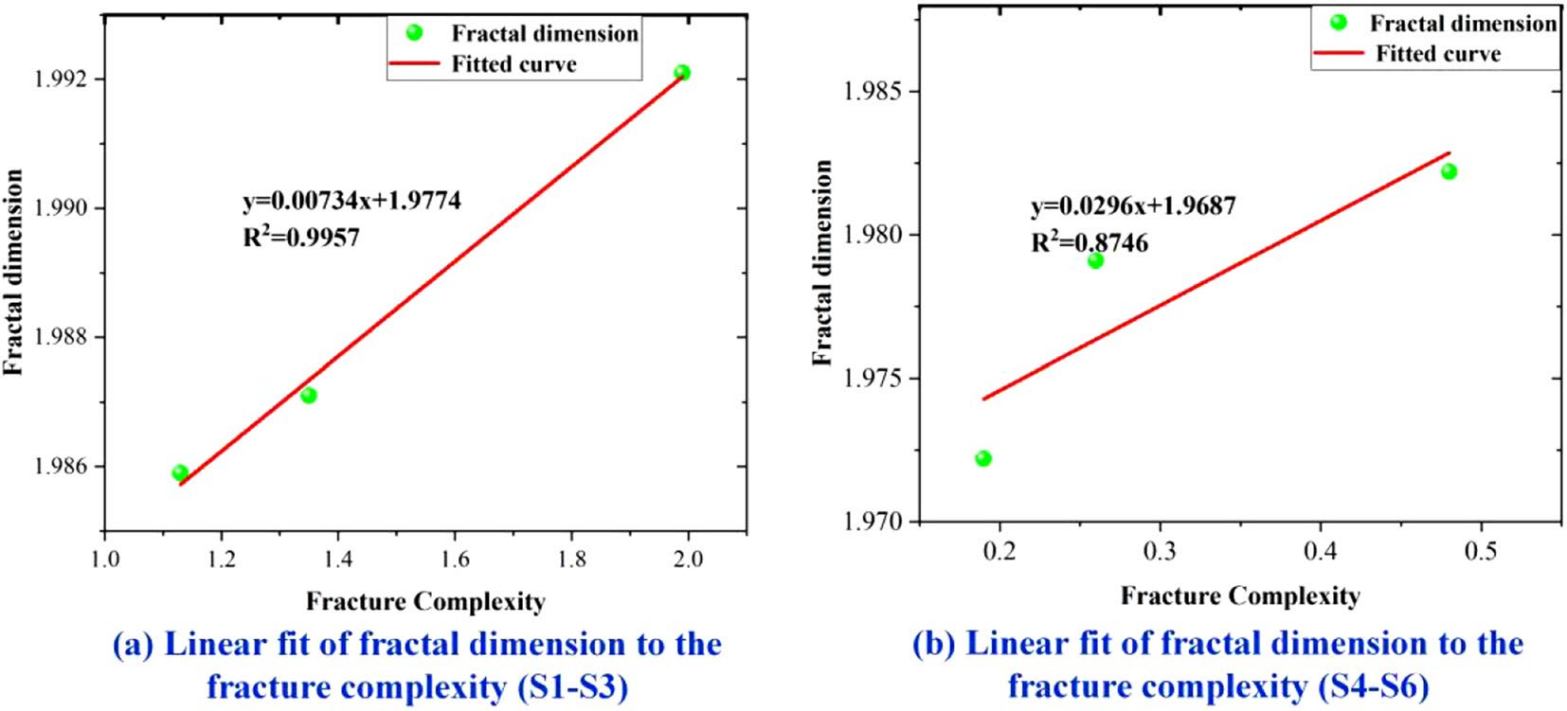
Linear fit of fractal dimension to fractal complexity.

Figure 12 shows the linear fit of fractal dimension and fracture complexity, and according to the fitting results, it was found that the fractal dimension was proportional to the fracture complexity. The correlation coefficients were 0.9957 and 0.8746. The fractal dimension was found to be a better indicator to describe the cleavage because it did not require complicated statistical work and contains more information compared with the cleavage complexity. The results of the experimental calculations for all specimens are summarized in Table 2.

**Table 2:** Experimental specimens: fracture complexity and fractal dimensions.

Specimen number	Tri-axial circumferential pressing	Relief pressure (MPa)	Fracture complexity	Fractal dimension
S1	σx = 4 MPa; σy = 7 MPa; σz = 5 MPa	50.54	1.99	1.9921
S2	σx = 4 MPa; σy = 7 MPa; σz = 5 MPa	38.34	1.35	1.9871
S3	σx = 4 MPa; σy = 7 MPa; σz = 5 MPa	27.11	1.13	1.9859
S4	σx = 4 MPa; σy = 7 MPa; σz = 5 MPa	40.13	0.48	1.9822
S5	σx = 4 MPa; σy = 7 MPa; σz = 5 MPa	36.86	0.26	1.9791
S6	σx = 4 MPa; σy = 7 MPa; σz = 5 MPa	25.60	0.19	1.9722

### Analysis of the principle of hydrothermal expansion and pressure degradation

Water in the normal state is generally considered to be an incompressible fluid. However, in this paper, the fluid was heated by the polyenergizer to make the fluid reach a high-temperature and high-pressure state. It was worth noting that the reaction temperature of the polyenergizer exceeded 1500 K. The short time heating must have given rise to an uneven temperature distribution, and the temperature near the polyenergizer was significantly higher than in other parts. Due to the constant pressure inside the system, the water density at the high temperature state was found to be lower according to the ideal gas equation of state. Ultrahigh-temperature and high-pressure water entered the fracture surface and expanded rapidly in an instant, which means that the fracture formation process could be considered as entailing almost no heat exchange between the fluid and the hole wall of the specimens. Therefore, this paper makes a reasonable assumption that the process was isentropic expansion process.

The thermodynamic law of the isentropic expansion process, is as follows:

3 $$\frac{{T_{2} }}{{T_{1} }} = \left( {\frac{{P_{2} }}{{P_{1} }}} \right)^{(k - 1)/k} = \left( {\frac{{V_{1} }}{{V_{2} }}} \right)^{k - 1}$$

where *T* is temperature K; *P* denotes pressure MPa; *V* is volume m^3^; *k* is the ratio *C*_p_/*C*_v_.

**Figure 13:**
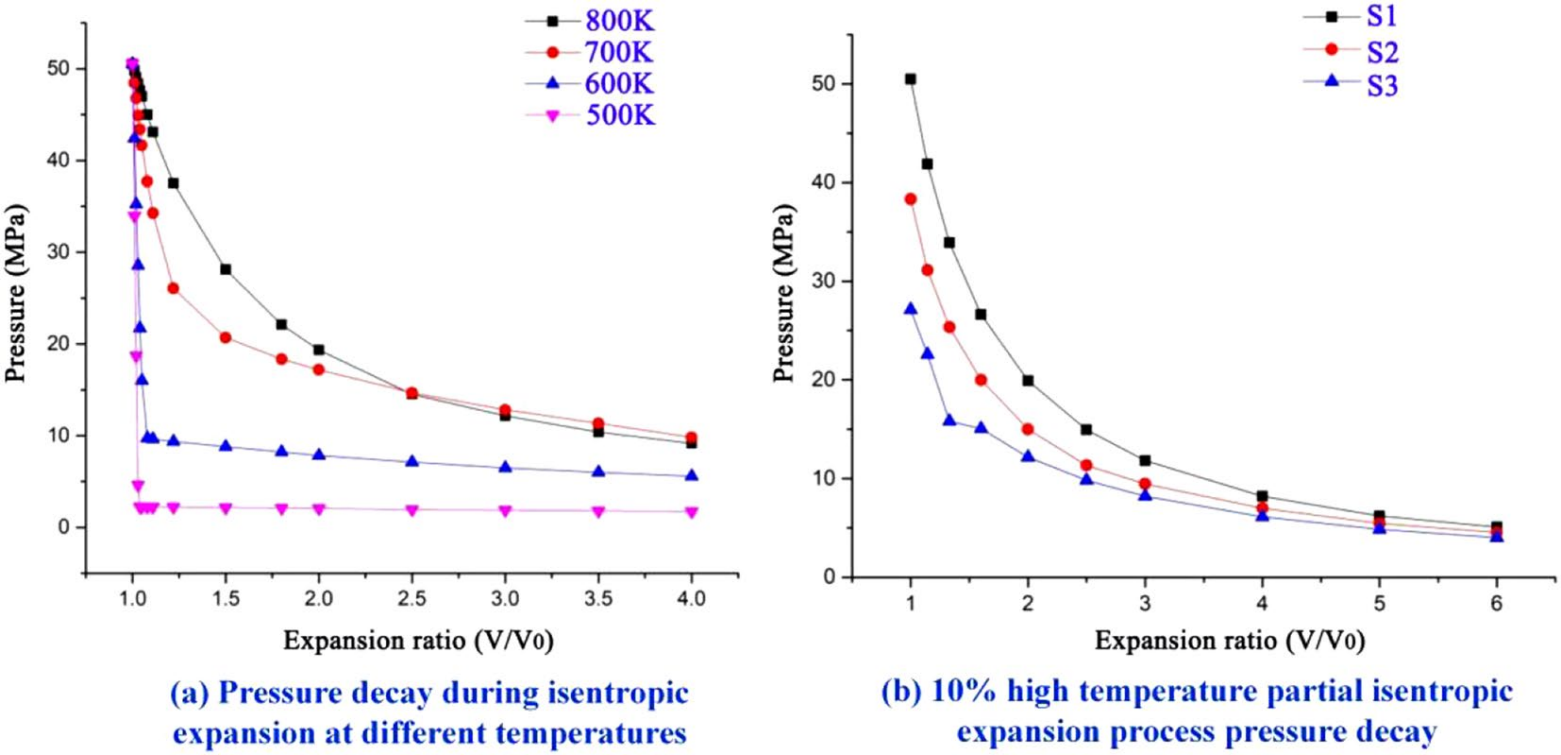
Pressure decay diagram under isentropic expansion.

In the present work, the pressure after the expansion of the isentropic process of water at different temperatures was analyzed, and it was found that the difference in the initial state temperature would cause a huge difference in the fluid pressure after expansion; the magnitude of fluid pressure directly decided whether the experimental fracturing and fracturing effect can be achieved. Therefore, the pressure decay of the isentropic expansion process was investigated at a pressure of 50.54 MPa and temperatures of 500 K, 600 K, 700 K, and 800 K. The thermodynamic parameters of water before isentropic expansion are shown in Table 3, and the specific pressure decay is depicted in Fig. 13. The water in the high temperature state is shown to have higher pressure under the same expansion ratio condition; the temperature reached 700 K and above can result in a higher expansion ratio, which is conducive to formation of a complex crack network. While in the isentropic expansion process at temperatures of 500 K and 600 K, the fluid quickly becomes a gas–liquid mixture, the pressure rapidly decayed below the tensile strength of the specimens, the corresponding seam-making ability must be significantly lower than the isentropic expansion process of higher temperature water.

**Table 3:** Initial parameters of isentropic expansion at different temperatures.

Temperature/K	Pressure/MPa	Density/(g/ml)	Entropy/(J/g*K)	Enthalpy/(J/K)
800	50.54	0.22	5.39	2890.3
700	50.54	0.49	4.29	2071.9
600	50.54	0.74	3.34	1454.8
500	50.54	0.87	2.50	991.9

The decay of pressure with the increase of expansion ratio (*V*/*V*_0_) for the isentropic expansion process of high-temperature and high-pressure water released at 50.54 MPa at different temperatures is shown in Fig. 13. The temperature of the polyenergy agent used in this experiment was higher than 1500 K. Super-high temperature water could be generated near the polyenergy agent during the heating of the water, and its ability to maintain the pressure of the isentropic expansion process was significantly higher than that of the part of water far from the polyenergy agent with lower temperature. After the impact cracking, the pressure was maintained at the level of 15–22 MPa, and it can be inferred that the ultrahigh temperature water plays a dominant role in the seam-formation process.

Through the above figure, for different temperatures of isentropic changes in expansion pressure, the expansion of water in the lower temperature state can be ignored, the density is considered as a constant; the isentropic expansion process was simplified to ultrahigh temperature part of the water expansion, pushing the lower temperature part of the water into the specimen fracture surface. Taking 50.54 MPa peak pressure release as an example, because the height of the heating agent tube was 20 mm, about one-tenth of the total height of the generator, so it was assumed that 10% of the fluid in the generator was in the high-temperature low-density region, the remaining 90% of the water remained in a lower temperature state. The parameters of the water in the whole system before the reaction were shown in Table 4. In addition, the polyenergy agent was loose porous material, and the volume after the reaction was neglected, thus obtaining the parameters of the water in the whole system before the reaction, and the parameters were obtained from the official website of the National Institute of Standards and Technique Research NIST.

**Table 4:** Fluid state parameters before and after heating.

Fluid environment	Density/(g/ml)	Pressure/MPa	Entropy/(J/g*K)
Before heating	0.917	0.10	0.66
High temperature part after heating	0.160	50.54	5.87
After heating high temperature part	0.160	38.34	5.56
After heating high temperature part	0.160	38.34	5.25

The high-temperature low-density part of the water isentropic expansion process of the temperature agent pressure decreased as shown in Fig. 13. The hypothetical conditions of the high-temperature low- density part of the expansion ratio of water were about 1.55–1.92 times. The theoretical analysis results and test results were better matched.

### Analysis of the principle of high-pressure subcritical water-impact fracturing

The pressure drop of fluids at the fracture surface was different from the static action process of hydraulic fracturing, and the traditional fracture expansion model of hydraulic fracturing was no longer applicable. Hence, the stress intensity factor theory of fracture mechanics was introduced in the present work to reveal the fracture-expansion mechanism of ultrahigh-temperature and high-pressure hydrothermal fracturing.

The problem was simplified to a two-dimensional problem, and the stress intensity factor of a type-I crack was analyzed assuming that the specimens was linearly elastic and homogeneous. The actual action of the stress system was complex, and the superposition principle was used, and the stress intensity factor at the crack tip was regarded as the superposition of the stress intensity factors formed by each simple load, and the expressions are shown in Eq. (4):

4 $$K_{I} (\sigma_{H} ,\sigma_{h,} p,p_{a} ) = K_{I} (\sigma_{H} ) + K_{I} (\sigma_{h} ) + K_{I} (p) + K_{I} (p_{a} )$$

where, $\sigma_{H}$ is the maximum horizontal principal stress;$\sigma_{h}$ is the minimum horizontal principal stress;$p$ represents the pressure case in the hole;$p_{a}$ is and the pressure distribution at the fracture face.

Paris and Sih^40^ provided a unified expression for calculating the stress intensity factor for an infinite flat plate crack length of 2*a*:

5 $$K_{I} = (\pi a)^{ - 1/2} \int_{ - a}^{a} {\sigma_{y} (x,0)\left( {\frac{a + x}{{a - x}}} \right)}^{1/2} dx$$

where: $K_{I}$ is the stress intensity factor;$\sigma_{y} (x,0)$ is the stress magnitude along the *y*-direction; $a$ is the crack half-length;$x$ denotes the crack coordinate.

The process of solving the stress intensity factor was to integrate the stress caused by the load as per Eq. (5). The stress distribution of the infinite perforated plate was known and the type of crack in this experiment was a type I open crack, caused by tangential positive stress. It was possible to solve to obtain and expression of stress intensity factor.

The solution was such that.

6 $$\sigma_{\theta } (x,0) = \frac{1}{2}\sigma_{H} \left[ {\left( \frac{R}{x} \right)^{2} - 3\left( \frac{R}{x} \right)^{4} } \right]$$

Furthermore,

7 $$K_{I} (\sigma_{H} ) = \frac{{\sigma_{H} }}{{2[\pi (R + a)]^{1/2} }}\int_{ - (R + a)}^{R + a} {\left[ {\left( \frac{R}{x} \right)^{2} - 3\left( \frac{R}{x} \right)^{4} } \right]\left( {\frac{R + a + x}{{R + a - x}}} \right)^{1/2} dx}$$

where $K_{I} (\sigma_{H} )$ is the stress intensity factor corresponding to the maximum horizontal principal stress;$R$ refers to the radius of the hole.

With the introduction of the dimensionless stress intensity factor coefficient *f*(*b*) and taking the positive value of the tensile stress for the convenience of calculation, the analytical solution of the stress intensity factor solution was obtained in the following equation. Rummel and Winter gave the analytical equation of the dimensionless stress intensity factor based on the conformal transformation of the complex variable function and the numerical approach of the boundary configuration.

8 $$K_{I} (\sigma_{H} ) = - \sigma_{H} \sqrt R f(b)$$

9 $$f(b) = - 2[(b^{2} - 1)/\pi b^{7} ]^{1/2}$$

Where, *b* is the normalized crack length coordinate; *f*(*b*) stands for the dimensionless intensity factor of the maximum horizontal principal stress.

Calculation and use of the dimensionless stress intensity factor in the same way gives:

10 $$\sigma_{y} (x,0) = \frac{1}{2}\sigma_{h} \left[ {2 + \left( \frac{R}{x} \right)^{2} + 3\left( \frac{R}{x} \right)^{4} } \right]$$

11 $$K_{I} (\sigma_{h} ) = - \sigma_{h} \sqrt R g(b)$$

12 $$g(b) = (\pi b)^{1/2} \left( {1 - \frac{2}{\pi }\sin^{ - 1} \frac{1}{b}} \right) + 2(b^{2} + 1)\left( {\frac{{b^{2} - 1}}{{\pi b^{7} }}} \right)^{1/2}$$

Where, *g*(*b*) is the dimensionless stress intensity factor of the minimum horizontal principal stress.

Considering the fluid pressure inside the fracture as zero, the solution yields the stress intensity factor due to the fluid in the hole is

13 $$K_{I} (p) = p\sqrt R h_{0} (b)$$

14 $$h_{0} (b) = 1.3\frac{b - 1}{{1 + b^{3/2} }} + 7.8\frac{\sin [(b - 1)/2]}{{2b^{5/2} - 1.7}}$$

Where $h_{0} (b)$ is the dimensionless strength factor corresponding to the pressure in the pore.

The stress intensity factor caused by the fluid acting on the fracture surface can be expressed similarly to that acting on the pore wall as:

15 $$K_{I} (p_{a} ) = p\sqrt R h_{a} (b)$$

However, there was a significant difference in the stress intensity factor caused by different pressure-drop gradients, which was reflected in the difference of the dimensionless stress intensity factor *h*_*a*_(*b*) in Eq. (15).

In the case of little change in crack cross-section, the crack volume during quasi-static expansion of the crack was linearly related, and the pressure-drop/volume-change relationship could be obtained from the above isentropic expansion curve of high-temperature and high-pressure water; the analysis found that the inverse pressure drop model corresponded well with the pressure drop gradient of the fluid expansion process. Therefore, the dimensionless stress intensity factor coefficient of fluid action on the crack face, using the inverse pressure gradient type, this gave:

16 $$p_{a} (x) = \left\{ \begin{gathered} - pR/x \,\,\,\,\,\,\,\,\,\,\,\, - (R + a) \le x \le - R \hfill \\ pR/x \,\,\,\,\,\,\,\,\,\,\,\,\,\,\,\,\, R \le x \le (R + a) \hfill \\ \end{gathered} \right.$$

The corresponding dimensionless stress intensity factor was:

17 $$h_{a} (b) = 2(\pi b)^{ - 1/2} \ln [b + (b^{2} - 1)^{1/2} ]$$

According to the theory of fracture mechanics and the stress intensity factor, for type-I cracks, when the stress intensity factor exceeded the fracture toughness, the cracks were extended. The crack extension criterion was:

18 $$p_{c} = \frac{1}{{h_{0} + h_{a} }}\left( {\frac{KIC}{{\sqrt R }} + \sigma_{H} + \sigma_{h} } \right)$$

**Figure 14:**
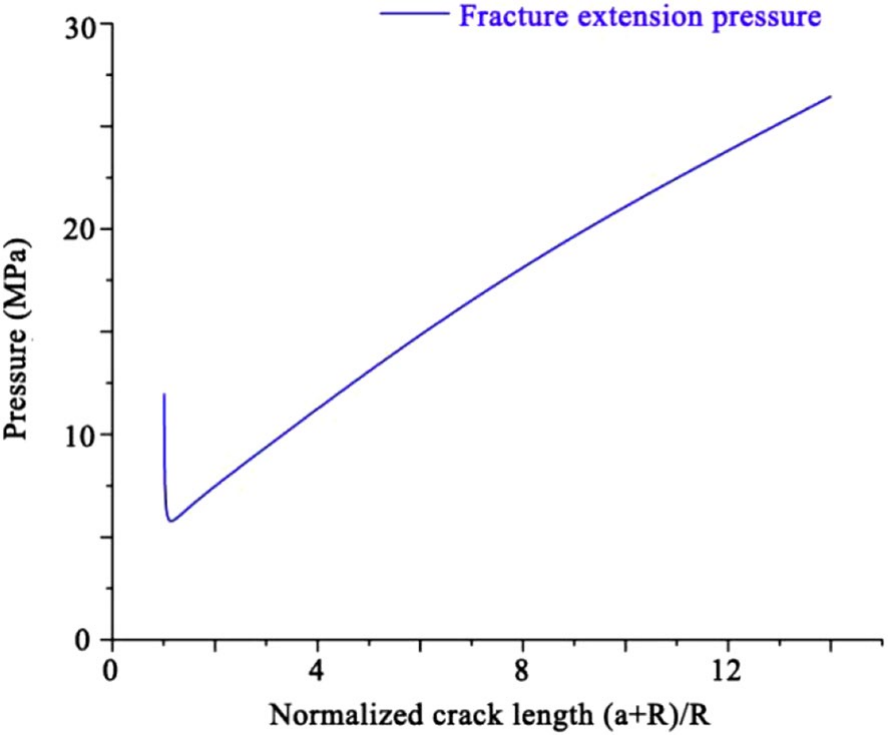
Variation of crack extension pressure with crack length.

The variation of the crack extension pressure with the initial crack length was obtained based on Eq. (18), and the plotted image, as a function of the crack length, is shown in Fig. 14.

The pressure required to extend the fracture increases as the initial fracture length increased, which can explain the higher pressure in the fracture extension phase after impact cracking in the high-impact-strength experimental group. Simultaneously, after the end of quasi-static fracture extension, this pressure gradient changed to a constant-type pressure gradient. The analysis found that the required intra-hole pressure was higher at this pressure gradient and fracture extension could not be achieved again. This also proved that high-temperature, high-pressure hydrothermal fracturing could achieve fracture extension at a lower fluid pressure. This model combined with the post-impact pressure scenario allowed for an explanation of the crack-extension-length scenario.

The specimens were not infinite and there was a size effect; and according to the energy minimum principle of crack extension, the crack length was not uniform in the extension of multiple cracks where the dominant crack extension inhibited the extension of other cracks. Therefore, the crack expansion was more complicated, and only the mechanism of crack expansion was reasonably explained herein.

### Analysis of pre-injection fracturing effect

The formation of radial cracks was caused by the high-pressure fluid applied to the hole wall, the positive radial stress caused tangential deformation, when the resulting positive tangential stress exceeded the tensile strength of the specimens, a radial crack was produced from the hole. The main reason for the formation of multiple cracks was that the high loading rate caused by the high rate of pressurization formed a non-static cracking effect. The number of radial fractures was related to the loading rate^41^. In combination with published data, the high-energy gas fracturing technique was usually controlled to produce between three and eight radial fractures in the reservoir to ensure the extraction efficiency; the pressurization rate ranged from 1 to 1000 MPa/ms.

19 $$t_{m} < \frac{{8\pi D_{w} }}{{C_{r} }}$$

where *C*_r_ denotes the surface wave velocity of the Rayleigh wave and *D*_w_ represents the diameter of the wellhead.

The model was similar to the pre-filled ultrahigh-pressure hydrothermal fracturing experiment, in which the fluid acted on the borehole wall; however, there were differences with the unfilled ultrahigh-pressure hydrothermal fracturing experiment, mainly in the unfilled model, in which the ultrahigh-pressure water vapor liquefied (phase change) after being ejected at the outlet when it was cold, and the pressure decayed dramatically, so that the pressurization rate at the outlet could not be regarded as the pressurization rate acting on the borehole wall. This was also consistent with the experimental phenomenon in the unfilled mode, where only three radial fractures were produced at the maximum pressurization rate of 8.74 MPa/ms; the main fractures at 5.04 MPa/ms and 0.87 MPam/s were all hydraulically-fractured. The number of radial fractures was positively correlated with the pressurization rate in the pre-filled mode; multiple radial main fractures were formed at high pressurization rates. In addition, the number of radial cracks was also related to the degree of specimen inhomogeneity, stress state and many other factors. Due to the small number of experiments and considering the pressurization rate as the only independent variable, the number of radial cracks tended to increase only with the pressurization rate, and no specific analytical formula or fitted relationship could be deduced.

In addition to the radial cracks, multiple branching cracks were observed on the free surface of the front face in three groups of experiments: pre-filled 50.54 MPa and 38.84 MPa and unfilled 40.13 MPa. Only a few of the multiple radial cracks were extended; this was due to the short crack-initiation time, short extension process, and anisotropy; the crack extension followed the principle of minimum energy, which promoted further extension of the longer cracks, making it difficult to extend the shorter ones. The high-temperature and high-pressure hydrothermal fracturing process was simplified into two stages: in the first stage, the ultrahigh-temperature and high-pressure water vapor impacted the liquid column (hole wall) instantaneously, and dynamic rupture occurred under the action of stress waves, forming between two and six radial fractures. The second stage was the quasi-static fracture extension stage driven by high-temperature and high-pressure water, in which high-temperature and high-pressure water vapor entered the fracture surface at high speed to achieve fracture extension. The final formation of cracking impact followed by the crack morphology of the specimens. Slick-water fracking resulted in simpler cracking and fewer cracks.

Two modes of ultrahigh-temperature and high-pressure hydrothermal experiments were designed, and the fracturing process of ultrahigh-temperature and high-pressure water in the two modes was slightly different. The high-temperature and high-pressure water vapor generated by the pre-filled UHP water fracturing experiment did not act directly on the specimens, but the expansion process first affected the liquid water column pre-filled in the gas circuit, and the pressure change transmitted by the liquid column caused cracking and crack extension in the specimens. For the water-filled mode of ultrahigh-temperature, high-pressure, hydrothermal fracturing experiments, the generator produced ultrahigh-temperature high-pressure water expansion through the gas path and directly on the specimen hole wall. The liquid column was considered as an incompressible fluid, and the difference between the two fracturing effects was mainly reflected in the difference of expansion ratio, as the pre-filled mode required a smaller expansion ratio and weaker pressure decay, so the fracturing effect was also better.

## Conclusion

A new high-pressure subcritical water impact fracturing technique was proposed, the true three-axis experimental system was independently designed, and six sets of experiments with pre-injection of water under different release pressure regimes were conducted. The following conclusions can be drawn:

1. The pressure rise in the reactor would be divided into five stages: initial reaction, linear pressure rise, rate decrease, instantaneous pressure release, and residual pressure stages;
2. Whether cracks were pre-filled with water or not mainly resulted in the difference of expansion ratio, pre-filled mode fracturing requires smaller expansion ratio, weaker pressure decay, and therefore a better fracturing effect;
3. As the initial fracture length increased, the pressure required to extend the fracture increased, high-pressure subcritical water impact fracturing can achieve fracture extension by lower fluid pressure;
4. The fractal dimension was proportional to the fracture complexity with a strong linear relationship, giving rise to a new option with which to evaluate the fracturing effect.

In the future, the temperature of the water would need to be measured, and the initial solution will be replaced with a different one to be analyzed in comparison with conventional fracking fluids such as slickwater.

## Data Availability

Te datasets generated during and/or analysed during the current study are available from the corresponding author on reasonable request.
